# Innovate! Accelerate! Evaluate! Harnessing the RE-AIM framework to examine the global dissemination of parenting resources during COVID-19 to more than 210 million people

**DOI:** 10.1186/s12889-024-19751-9

**Published:** 2024-09-03

**Authors:** Jamie M. Lachman, Nisso Nurova, Angelique Nicole Chetty, Zuyi Fang, Alison Swartz, Lorraine Sherr, Helen Mebrahtu, Kasonde Mwaba, Ohad Green, Isang Awah, Yuanling Chen, Inge Vallance, Lucie Cluver

**Affiliations:** 1 Department of Social Policy and Intervention, University of Oxford, Oxford, Barnett House, 32 Wellington Square, OX1 2ER UK; 2 Centre for Social Science Research, University of Cape Town, Cape Town, South Africa; 3 Social and Public Health Sciences Unit, University of Glasgow, Glasgow, UK; 4 Parenting for Lifelong Health, Oxford, UK; 5 Institute of Population Research, Peking University, Beijing, China; 6 Institute of Medical and Biomedical Education, St George’s University of London, London, UK; 7 Division of Social and Behavioural Sciences, School of Public Health and Family Medicine, University of Cape Town, Cape Town, South Africa; 8 Institute for Global Health, University College London, London, UK; 9 Department of Psychiatry and Mental Health, University of Cape Town, Cape Town, South Africa

**Keywords:** RE-AIM framework, Implementation science, Parenting, COVID-19

## Abstract

**Background:**

Parents were at the forefront of responding to the needs of children during the COVID-19 pandemic. This study used the RE-AIM framework to examine the Reach, Effectiveness, Adoption, Implementation, and Maintenance of a global inter-agency initiative that adapted evidence-based parenting programs to provide immediate support to parents.

**Methods:**

Data were collected via short surveys sent via email, online surveys, and analysis of social media metrics and Google Analytics. Retrospective surveys with 1,303 parents and caregivers in 11 countries examined impacts of the resources on child maltreatment, positive relationship building, parenting efficacy, and parenting stress.

**Results:**

The parenting resources were translated into over 135 languages and dialects; reached an estimated minimum 212.4 million people by June 2022; were adopted by 697 agencies, organizations, and individuals; and were included in 43 national government COVID-19 responses. Dissemination via social media had the highest reach (*n* = 144,202,170, 67.9%), followed by radio broadcasts (*n* = 32,298,525, 15.2%), text messages (*n* = 13,565,780, 6.4%), and caseworker phone calls or visits (*n* = 8,074,787, 3.8%). Retrospective surveys showed increased parental engagement and play, parenting self-efficacy, confidence in protecting children from sexual abuse, and capacity to cope with stress, as well as decreased physical and emotional abuse. Forty-four organizations who responded to follow-up surveys in April 2021 reported sustained use of the resources as part of existing services and other crisis responses.

**Conclusion:**

This study highlights the importance of a) establishing an international collaboration to rapidly adapt and disseminate evidence-based content into easily accessible resources that are relevant to the needs of parents; b) creating open-source and agile delivery models that are responsive to local contexts and receptive to further adaptation; and c) using the best methods available to evaluate a rapidly deployed global emergency response in real-time. Further research is recommended to empirically establish the evidence of effectiveness and maintenance of these parenting innovations.

## Background

The SARS-CoVCoV-2 (COVID-19) pandemic negatively impacted the lives of children and families globally, with increased risks for those living in low- and middle-income countries (LMICs) or marginalized communities [1]. School closures affecting approximately 1.4 billion children worldwide at the height of the pandemic resulted in substantial developmental and learning loss [2]. At a time when there was an increased need for support, parents often found themselves solely responsible for the care of their children without access to social networks and services. Increased rates of parenting stress, financial insecurity, and exposure to harmful online content led to dramatically increased risks of violence against children and intimate partner violence [3]. A meta-analysis found that the global prevalence of child and adolescent depression and anxiety doubled during COVID-19 in comparison with pre-pandemic estimates [4]. An additional 10.4 million children were affected by orphanhood and caregiver morbidity due to COVID-19 [5].

Movement restrictions prohibiting face-to-face interactions during COVID-19-related lockdowns forced public health and social service providers to adapt normal service delivery approaches. Although there is considerable evidence demonstrating the effectiveness of face-to-face parenting programs based on social learning and attachment theories [6, 7], most of these programs were developed for in-person group- or home-based delivery and were thus not feasible during the pandemic. This led to a proliferation of alternative delivery approaches to provide parenting support, including via telehealth, social media, digital apps, online groups, chatbots, telephone consultations, and other remote methods [8–10].

Emerging evidence supports the potential effectiveness of remotely delivered parenting interventions using online platforms, apps, and chatbots on improving parent–child interaction and child development outcomes [11, 12]. Additional research supports the efficacy of parenting programs delivered via radio and television [13], and there is limited but promising evidence for population-level delivery of parenting support [14]. Moreover, two recent studies showed promising results on improving family functioning and reducing child behavior problems through a light-touch parenting intervention using leaflets and informal group conversations with families in the West Bank and Indonesia [15, 16].

In response to the increasing risks of violence against children and reduced capacity to provide in-person parenting support during COVID-19, Parenting for Lifelong Health (PLH), a UK-based charity, led an interagency emergency response effort – the COVID-19 Playful Parenting Emergency Response – to rapidly adapt evidence-based parenting programs into freely available, open-source, and culturally sensitive resources. These evidence-informed resources were initially released for public use via the World Health Organization (WHO) and UNICEF COVID-19 websites, alongside a letter in The Lancet [17]. The resources focused on building positive parent–child relationships and reducing violence against children by encouraging caregiver playful engagement, reinforcing positive child behaviors managing child difficult behaviors, creating daily structure and routines, talking about COVID-19, keeping children safe online, and reducing caregiver stress and family conflict.

The purpose of this study was to examine the global dissemination of these parenting resources during the height of the COVID-19 pandemic. We used the RE-AIM to examine 1) the adoption of the resources by implementing agencies, including government and non-governmental organizations (NGOs); 2) their delivery by implementing agencies; 3) the reach, or number of people who received the resources across geographical areas and dissemination methods; 4) their impact on positive parenting, parenting stress, parent efficacy, and child physical and emotional abuse; and 5) their long-term maintenance and use beyond the pandemic [18].

## Methods

This real-world study was conducted remotely from March 2020 to July 2021. It was conducted in parallel to a qualitative study examining perceptions of parents and service providers on the usefulness and appropriateness of the parenting resources [19]. Ethical approval was granted by the University of Oxford (Ref #R69569) and University of Cape Town (Ref# PSY2021-038).

### RE-AIM framework

The RE-AIM framework is one of the most widely used implementation science frameworks to support the understanding of how evidence-based behavioral and public health interventions are integrated into policy and practice [20]. It allows for the assessment of multiple implementation science domains across different ecological levels (i.e., individual, organizational, national, etc.). RE-AIM dimensions include 1) Reach: the number, proportion, and representativeness of individuals who participate in a given intervention; 2) Effectiveness: the impact of a given intervention on primary and secondary outcomes as well as potential variability based on population characteristics; 3) Adoption: the level of take up and representativeness by implementers; 4) Implementation: the fidelity and quality of delivery, as well as adaptation, of an evidence-based intervention, particularly as it is delivered in different contexts and cultures; and 5) Maintenance: the extent to which an intervention becomes institutionalized through sustained delivery and the sustainment of effects on an individual level [21]. In this study, we adapted the sequencing of RE-AIM to provide further understanding of the global scale-up and impact of the COVID-19 Parenting resources. First, we examined the *Adoption* and *Implementation* by implementing partners, governments, and other agencies. Second, we examined the *Reach* of the COVID-19 Parenting resources. Third, we assessed the *Effectiveness*, or impact, of the resources on behavioral and mental health outcomes in a multi-country study using retrospective surveys. Finally, we assessed the *Maintenance* of resources by implementing partners one-year after initial dissemination (see Table 1).

**Table 1:** Application of RE-AIM to evaluate COVID-19 parenting resources

Dimensions of RE-AIM	Operational definition	Outcomes	Data level	Data sources
Adoption	Absolute number, type, and geographic diversity of agencies who used the COVID-19 Parenting resources; Exclusion criteria: No evidence of uptake	Absolute number and types of agencies	Organizational	Emails, online surveys, social media metrics, Google Analytics, and direct distribution
Implementation	Methods of delivery and adaptation of resources by implementing agencies	Delivery modality; level of fidelity; adaptation	Organizational	Emails, online surveys, social media metrics, Google Analytics, and direct distribution
Reach	Absolute number of people reached with parenting resources, disaggregated by geographical location and dissemination method	Total number; number by geographical area; number by dissemination method	Individual	Emails, online surveys, social media metrics, Google Analytics, and direct distribution
Effectiveness	Impact of COVID-19 Parenting resources on behavioral and mental health outcomes	Playful parenting, positive reinforcement, parent self-efficacy to protect children from sexual abuse, parenting stress, physical abuse, emotional abuse	Individual	Retrospective surveys conducted in 11 countries
Maintenance	Extent to which an intervention becomes institutionalized on an organizational level	Use and adaptation of resources 1-year after initial dissemination	Organizational	Open-ended short survey, reports from implementing agencies

### COVID-19 playful parenting resources

**Fig. 1:**
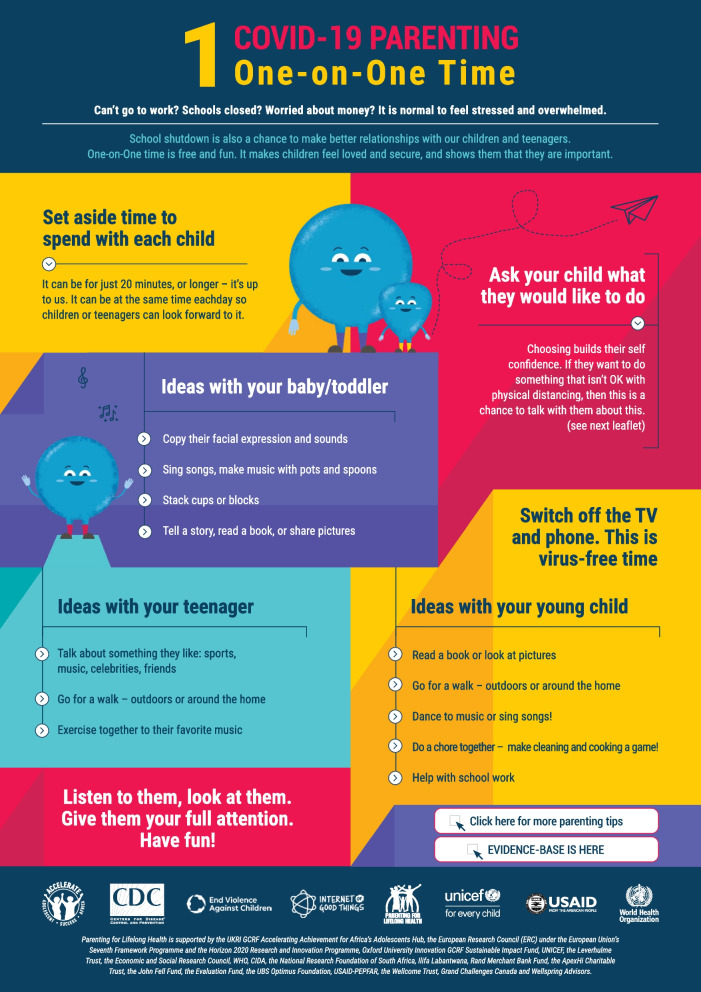
Example of tip sheet design

The COVID-19 Playful Parenting Resources were developed in March 2020 by adapting core parenting themes from the Parenting for Lifelong Health suite of parenting programs originally developed and tested in LMICs [22]. Six initial single-sided tip sheets were created: 1) positive relationship building, 2) positive reinforcement, 3) limit setting, 4) managing misbehavior, 5) coping with stress, and 6) talking about COVID-19. In April 2020, an additional six tip sheets were developed: 7) online child safety, 8) family budgeting, 9) intimate partner relationships, 10) remote educational support, 11) learning through play, and 12) anger management. Specific sheets were also created for families living in crowded homes and communities, those with disabilities, adolescents, and newborns (see Table 2). Each tip sheet distilled evidence-based parenting content into three to four core skills with an overall limit of 150 to 200 words per sheet. Gender-neutral comic characters were developed as part of the design process to increase user-friendliness. The content was framed positively (e.g., “Set aside time to spend with each child”) with empathy towards the current experiences of parents and caregivers during COVID-19 (e.g., “It is normal to feel stressed and overwhelmed”). Core evidence-based parenting content was also contextualized for COVID-19, such as suggested activities for child-led play during lockdown and specific routines around safe distancing and hygiene. Hyperlinks were also included for additional support as well as to UNICEF and WHO COVID-19 websites (see Fig. 1).

**Table 2:** COVID-19 playful parenting tip sheet topics and parenting skills

**Set**	**Title**	**Parenting skill**
Original Tip Sheets	One-on-One Time	Positive relationship building
	Keeping It Positive	Praise and positive instructions
	Structure Up	Rules and routines
	When Children Misbehave	Positive discipline
	Keep Calm and Manage Stress	Stress reduction
	Talking about COVID-19	Risk reduction and emotional regulation
Additional Tip Sheets	Keeping Children Safe Online	Online child safety and digital parenting
	Family Budgeting in Times of Financial Stress	Creating family budgets and savings plans
	Family Harmony at Home	Intimate partner negotiation and nonviolent conflict resolution
	Learning through Play	Playful parenting and child development
	Education and Remote Learning	Parent engagement and support of education
	When We Get Angry	Stress reduction and emotional self-regulation
Tip Sheets for Specific Target Groups	Parenting in Crowded Homes and Communities	Child safety, hygiene, physical distancing, co-parenting, physical exercise, stress reduction
	Tips for Children with Disabilities	Disabilities inclusion and safety
	Tips for Parenting Teens	Parenting with adolescents
	Parenting a New Baby	Parenting with newborns

Prior to public release, the resources underwent a rigorous review process with core institutional partners. These included representatives from UNICEF, WHO, USAID, UNODC, the Global Partnership to End Violence Against Children, Clowns Without Borders South Africa, the Early Childhood Development Action Network, World Without Orphans, the Centers for Disease Control and Prevention, and the World Childhood Foundation. Subsequent translation into 135 languages and dialects involved 192 volunteers recruited primarily via Facebook, with additional support from World Without Orphans, Translators Without Borders, and volunteers from Generali Insurance and The Human Safety Net. The resources were uploaded to a central website depository (www.covid19parenting.com) and licensed under a Creative Commons Attribution-NonCommercial-ShareAlike 4.0 International license (CC BY-NC-SA 4.0). Creative Commons licensing was selected to allow for the open-source adaptation and replication of the resources freely without any restrictions or payment. In addition, a COVID-19 Playful Parenting dissemination team was established to support the reproduction of the tip sheets into social media toolkits, toolkits for social workers, and audio guides for radio and public service announcements.

### Data collection

Data on the adoption, implementation, and reach of the COVID-19 Parenting resources were collected from a total of 697 implementing agencies across 198 countries and territories. Research assistants based between the United Kingdom and South Africa collected data remotely through email correspondence, online surveys, and from social media platforms and Google Analytics. Individuals from 568 organizations who had signed up on the COVID-19 website mailing list or attended regional webinars hosted by UNICEF and the WHO were initially contacted via email requesting information about their dissemination of the resources. Email messages invited them to report either directly via email or via a brief online survey. Those who reported via email were asked to respond to two questions: “How have you shared the COVID-19 Parenting tips?” and “How many people have you reached?” Research assistants then followed up with respondents via email to collect more detailed data on the modality of delivery. The online survey consisting of the following items: 1) a multiple-select question about the modality of delivery, 2) a numeric question on the approximate reach for each selected modality, 3) yes/no question about whether they made any changes or adaptations to the original COVID-19 Parenting tip sheets, 4) an open-ended follow-up question on how they changed the resources, 5) an open-ended question asking whether they received any feedback on the impact or usefulness of the resources, 6) an open-ended question requesting suggestions for improving the content and delivery of the resources, 7) a yes/no question about whether they knew of any other organizations sharing the resources, 8) referral contact details for these organizations, and 9) an invitation to participate in in-depth interviews on their work supporting parents and children during the pandemic (for more information regarding the qualitative component, please see [19]).

We received a total of 265 responses (46.7%) of which 195 were received directly via email (73.6%) and an additional 60 via the online survey (26.4%). Data from a further 129 implementing agencies were also collected by analyzing social media metrics from Twitter, Facebook, LinkedIn, and Instagram and Google Analytics of selected COVID-19 response websites. Research assistants conducted online searches for evidence of uptake, implementation, and reach using key terms and hashtags (e.g., #COVID19parenting). Key implementing partners such as UNICEF and the WHO also reported the number of downloads of the parenting resources using Google Analytics, which was also monitored on the project’s own website (www.covid19parenting.com).

Implementing agencies that responded to the initial surveys were sent a follow-up email in which they were invited to participate in a retrospective study with families in their networks who received the COVID-19 Parenting resources. Thirty-four responses out of 265 potential organizations were received (12.8% response rate), from which 12 organizations volunteered to participate in the (35.2% recruitment rate). These organizations were from 11 countries, including five from Africa (Cameroon, Ghana, Malawi, South Africa, and Zambia), five in Asia (Cambodia, India, Nepal, Pakistan, and Sri Lanka), and one in Europe (North Macedonia). Parent and caregiver respondents (*N* = 1,303) were primarily recruited by implementing agencies through existing services offering a range of material and psychosocial support during the pandemic. Surveys were administered in a variety of formats, including phone-based interviewing by implementing staff, survey links on organization websites and social media pages, and home visits during provision of other services.

The retrospective survey was designed to be low-cost and simple to administer due to limited human and financial resources available as well as movement restrictions during the pandemic. It included four items on demographic characteristics (i.e., gender, age, number of children, age group of children), two items on method of delivery and type of content delivered (i.e., “Please tell us how you received the COVID-19 parenting messages” and “Which COVID-19 resources did you learn from?”), and six items examining their perceptions of the impact of the parenting resources on their behavior and mental health. These items were derived from psychometrically tested scales and included parent engagement in play, parenting stress, physical child abuse, emotional child abuse, positive parenting self-efficacy, and parent self-efficacy to protect their children from sexual abuse. Respondents were asked to reflect on the past month after receiving the parenting resources and to indicate on a Likert scale whether they agreed or disagreed with each statement (0 = Strongly disagree; 4 = Strongly agree). Variables were converted to binary variables, with ‘strongly agree’ and ‘agree’ grouped as ‘agree,’ and ‘neutral,’ ‘disagree,’ and ‘strongly disagree’ grouped as ‘disagree’. Lastly, respondents were asked an open-ended question about how they used the parenting resources. Data was uploaded securely to a dedicated study site on www.onlinesurveys.co.uk. All quantitative data were fully anonymous and delinked from IP addresses (Table 3).

**Table 3:** Outcomes used in retrospective surveys

**Outcome**	**Question**	**Measure**
Playful parenting	I am spending more time playing with my children or doing other fun activities together	Parenting Young Children, positive parenting subscale [23]
Parenting stress	I am more able to manage my stress as a parent/caregiver	Parenting Stress Scale [24]
Physical abuse	I am using less physical discipline like hitting, spanking, or slapping	ISPCAN Child Abuse Screening Tool-Trial, physical abuse subscale [25]
Emotional abuse	I am shouting, yelling, or screaming at my children less often	ISPCAN Child Abuse Screening Tool-Trial, emotional abuse subscale [25]
Parent self-efficacy	I feel more confident about using what I learned to have a positive relationship with my children	Parenting Sense of Competence, self-efficacy subscale [26]
Protection from sexual abuse	I feel more confident about protecting my children from online or in-person sexual abuse	Parental Discussion of Child Sexual Abuse [27]

Additional qualitative data were collected via in-depth interviews with parents, adolescent children, and providers (*n* = 22) from 14 countries and one global source. These interviews were conducted online using Zoom software, lasted an average of 22.3 min (range 7–46 min), and followed a semi-structured protocol. Qualitative data was analyzed using thematic analysis and is reported in detail in [19].

In April 2021, a final maintenance survey was sent via email to the implementing partners who had previously reported using the COVID-19 Parenting Resources. The surveys consisted of three questions: 1) Is your organization still using the COVID-19 Parenting Resources? If so, how are you using them? 2) Have you made any changes/adaptations to the resources that we provided? and 3) Is there any additional content we can assist with due to the emerging issues/needs your organization is currently facing? Only 44 out of 265 (16.6%) implementing agencies who initial reported using the COVID-19 parenting resources responded to these maintenance surveys one-year after initial dissemination.

### Data analyses

Data analyses used a pragmatic approach based on FUPS principles (i.e., using flawed, uncertain, proximate, and sparse data) [28] given the study’s limited financial and human resources.

### Adoption

Adoption by implementing agencies was assessed by examining the number and type of organizations that either reported on the delivery of the COVID-19 Parenting resources or were identified during social media platform searches. The type of adopting organization was disaggregated into seven categories: government, international NGO, local NGO, media, faith-based, academia, and individual.

### Implementation

Implementation data was analyzed based on the type of delivery modality, number of languages the resources were translated into, and the geographical representation of implementing partners.

### Reach

We used a conservative approach to establish an estimate for the absolute number of people who may have received the parenting resources between March 2020 and July 2021. This approach followed recommendations from the UNICEF guidance on Risk Communication and Community Engagement Indicators for COVID-19 Global Response [29]. If an implementing agency disseminated the resources across multiple social media platforms, we only included the platform with the highest reach to avoid double counting. Dissemination via the radio was estimated using available data from average listenership for the show that was broadcasting the resources. Otherwise, average hourly listenership was estimated based on radio station reports. Reach was disaggregated by organization type, geography, and dissemination methods (i.e., website visits/downloads, emails, social media, text messages, print media, radio, public service announcements, video, webinars, school-based, phone-based, faith-based, through case workers, or ‘other’). We also disaggregated reach via social media by the type of platform (i.e., Twitter, Facebook, Instagram, LinkedIn) and the quality of reach, or engagement, based on the number of likes, comments, and shares per platform.

### Effectiveness

Data from retrospective surveys were analyzed using RStudio (version 4.0.4). Descriptive statistics (i.e., numbers and percentages for categorical variables; means and standard deviations for continuous variables) examined demographic characteristics (i.e., adult gender, adult age, number of children in the household, child age group, source of information, and tips received) and behavioral and mental health outcomes (i.e., parent–child play, parental stress management, physical abuse, emotional abuse, and sexual violence protection). Chi-square tests were conducted to explore differences by gender (significant level *p* < 0.05).

Multivariate logistic regressions investigated potential factors associated with each behavioral or mental health outcome using odds rations (OR). This was done by first controlling for three participant-level categorical variables (adult gender, adult age, and number of children) and then adding in the source of information (e.g., type of sources or number of sources). When appropriate, generalized linear mixed models (GLMMs) were employed to account for clustering effects, with all variables used in the logistic regression being the fixed effects and the project/country variable being the random effect. Random-intercept, fixed-slope models were used with the assumption that people in the same organization or country exhibited the same relationships between the outcomes and demographic characteristics and that the intercept was allowed to vary for each level of the random effects. Variations in the time between receiving resources and responding to the surveys were accounted for.

### Maintenance

Responses from the maintenance survey conducted in April 2021 were analyzed to determine the method of dissemination, whether any adaptations had occurred, and recommendations for future adaptation. We also ran online searches on social media platforms to determine whether any organizations were still using social media to disseminate the resources.

## Results

### Adoption

Data analysis of adoption identified 697 implementing agencies, organizations, and individuals who disseminated the COVID-19 Playful Parenting resources. Direct dissemination by individuals on their personal social media platforms accounted for 33.0% of known adopters (*n* = 230). Out of the remaining 467 implementing agencies and organizations, the majority were NGOs, (e.g., Rwanda Refugee Camps, *n* = 187), followed by international agencies (e.g., UNICEF country offices, *n* = 93), faith-based organizations (e.g., Catholic Relief Services, *n* = 48), media-based organizations (*n* = 46), government agencies (*n* = 43), academic institutions (*n* = 39), and the private sector (*n* = 12) (see Table 4 for adoption by organization type).

**Table 4:** Adoption by organization type

**Organization type**	**N of organizations; %**	**N of reach; %**
NGO	187; 26.8%	9,678,991; 4.6%
International agency	93; 13.3%	144,718,130; 68.1%
Faith-based	48; 6.9%	20,938,016137; 9.9%
Government agency	43; 6.2%	6,774,803; 3.2%
Academic	39; 5.6%	5,236,279; 2.5%
Media	46; 6.6%	13,073,552; 6.2%
Private sector	12; 1.7%	10,288,892; 4.8%
Individual	230; 33.0%	1,746,810; 0.8%
**Total**	**698**	**212,429,326**

Adoption by region included 142 in Africa, 135 in Europe, 92 in Asia, 54 in North America, 47 in Latin America and the Caribbean, and 18 in the Middle East and North Africa. A further 210 organizations and individuals were either global or unspecified. Forty-three government agencies in 34 countries incorporated the COVID-19 Parenting resources into their national responses; twelve were in Asia, nine in Latin America and the Caribbean, seven in Europe, and six in Africa. Adopting government agencies included ministries of health, social development, education, and multi-agency COVID-19 responses.

### Implementation

The COVID-19 Parenting resources were adapted into a variety of dissemination methods. These included tip sheets for social worker case management, public service announcements, audio packs with scripts for radio announcements, videos shared on YouTube and other platforms, social media kits, faith-based guides for church and mosque leaders, and a song written and performed by a Broadway songwriter and producer which was rerecorded by individuals in Brazil, France, and Kenya. Out of a total of 939 implementation instances reported, the most common implementation method was social media (*n* = 567, 61.3%), followed by webinars (*n* = 60, 6.4%), print media (*n* = 50, 5.3%), websites (*n* = 47, 5.0%), email (*n* = 39, 4.2%), text messages (*n* = 39, 4.2%), and radio (*n* = 34, 3.6%) (see Table 5 for implementation by dissemination modality).

**Table 5:** Implementation by dissemination modality

**Dissemination type**	**N of implementers**^a^**; %**	**N of reach; %**
Social media	576; 61.3%	144,205,170; 67.6%
Website	47; 5.0%	1,434,229; 0.7%
Print media	50; 5.3%	465,180; 0.2%
Email	39; 4.2%	54,728; 0.0%
Texts	39; 4.2%	13,565,780; 6.4%
Radio	34; 3.6%	32,298,525; 15.2%
Video	18; 1.9%	2,386,063; 1.1%
Webinar	60; 6.4%	42,388; 0.0%
Public service announcements	5; 0.5%	5,028,708; 2.4%
School-based	9; 1.0%	91,900; 0.0%
Caseworkers	20; 2.1%	8,074,787; 3.8%
Religious leaders	13; 1.4%	3,165,836; 1.5%
Other	24; 2.6%	1,551,319; 0.7%
**Total**	**939**	**212,425,618**

^a^Number of organizations and individuals reporting using specific dissemination method

### Reach

Based on a conservative approach to establish an estimate for the absolute number of people reached (i.e., only counting one social media platform per implementer), analyses show that 212,425,618 people received the resources between March 2020 and July 2021. Global estimates that could not be attributed to a specific region or country accounted for 42.3% of those reached (*n* = 89,917,490). Adopters in Asia represented the highest reach by region (*n* = 59,265,444; 27.9%), followed by Africa (*n* = 32,104,123; 15.1%), Europe (*n* = 17,487,143; 8.2%), Latin America and the Caribbean (*n* = 8,498,905; 4.0%), and the Middle East and North African region (*n* = 3,912,399; 1.8%). North America accounted for the lowest reach by geographical region (*n* = 1,240,114; 0.6%) (Table 6).

**Table 6:** Population reach by geographical region

**Region**	**Reach**	**%**
Global	89,917,490	42.3%
Africa	32,104,123	15.1%
Asia	59,265,444	27.9%
Europe	17,487,143	8.2%
Latin America and Caribbean	8,498,905	4.0%
Middle East and North Africa	3,912,399	1.8%
North America	1,240,114	0.6%
**Total**	**212,425,618**	**100%**

Social media had the highest reach (*n* = 144,202,170, 67.9%), followed by radio (*n* = 32,298,525), 15.2%), text messages (*n* = 13,565,780, 6.4%), caseworker phone calls or visits (*n* = 8,074,787, 3.8%), public service announcements (*n* = 5,028,708, 2.4%), religious leaders (*n* = 3,165,836, 1.5%), and videos (*n* = 2,386,063, 1.1%) (Table 5). Disaggregating reach by social media platform and quality of reach, Facebook had the highest reach and engagement (reach: 135,988,014; engagement: 20,743), followed by Twitter (reach: 19,405,657; engagement: 829), Instagram (reach: 13,484,594; engagement: 5,704), and LinkedIn (reach: 4,758,985; engagement: 16) (Table 7).

**Table 7:** Breakdown reach and engagement through social media

Social media platform	Total reach	Engagement
Facebook	135,988,014	20,743 (likes, comments, shares)
Twitter	19,405,657	829 (likes, replies, retweets)
Instagram	13,484,594	5,704 (likes, comments)
LinkedIn	4,758,985	16 (likes, comments)
**Total**	**144,205,878**	**219,702 (likes, comments, replies, or shares**

### Effectiveness

#### Sample demographics

Online retrospective surveys were completed by 1,303 caregivers from 12 implementing agencies, with the number of respondents ranging from 12 to 241 per agency. Of the respondents, 949 (72.8%) were female. The mean caregiver age was 40.37 years (SD 10.47; 17-86). In terms of child age, 15.8% (*n* = 217) of the caregivers had an infant or toddler aged 0-23 months*;* 58.5% (*n* = 802) of them had a young child between 2-9 years; and 64.0% (*n* = 877) had a teenager aged 10-17 years. One-fifth of the respondents had four or more children in the household (20.6%, *n* = 283) (Table 8).

**Table 8:** Characteristics of retrospective survey respondents by implementing agency

**Agency**	**Country**	**Type**	**N**	**Female,** **N (%)**^a^	**Caregiver age,** **M (SD)**	**N of children, M (SD)**^b^	**Child age groups**			
							**0-23 Months,** **N (%)**	**2-9 Years,** **N (%)**	**10-17 Years,** **N (%)**	**Multiple ages,** **N (%)**
Gabriel Project Mumbai	India	NGO	166	74 (44.6)	36.31 (6.03)	3.11 (1.33)	11 (6.6)	124 (74.7)	119 (71.7)	86 (51.8)
Forgotten Voices International	Zambia	FBO	241	180 (76.3)	46.76 (11.93)	5.05 (2.10)	44 (18.3)	146 (60.6)	186 (77.2)	113 (46.9)
Forgotten Voices International	Malawi	FBO	39	20 (48.7)	44.11 (11.02)	4.66 (2.25)	13 (33.3)	29 (74.4)	29 (74.4)	26 (66.7)
Alternativa	North Macedonia	NGO	57	56 (98.2)	39.04 (5.88)	1.77 (0.76)	7 (12.3)	45 (78.9)	19 (33.3)	14 (24.6)
Karkhana	Nepal	NGO	12	10 (83.3)	37.00 (3.72)	2.25 (1.64)	0 (0.0)	8 (66.7)	7 (58.3)	3 (25.0)
Society for Promotion of Initiatives in Sustainable Development	Cameroon	NGO	129	86 (67.2)	39.07 (7.67)	3.55 (1.34)	41 (31.8)	102 (79.1)	96 (74.4)	83 (64.3)
Catholic Health Commission	Malawi	FBO	120	110 (93.2)	43.35 (10.60)	4.09 (1.56)	2 (1.7)	6 (5.0)	114 (95.0)	2 (1.7)
Alliance Development Trust	Sri Lanka	NGO	234	203 (87.1)	39.01 (9.92)	2.21 (0.98)	35 (15.0)	131 (56.0)	178 (76.1)	100 (42.7)
Protection and Help of Children Against Abuse Network (PACHAAN)	Pakistan	NGO	32	15 (51.7)	36.03 (7.85)	N/A	7 (21.9)	18 (56.3)	7 (21.9)	0 (0.0)
Save the Children	South Africa	NGO	95	68 (71.6)	35.81 (7.56)	2.22 (1.35)	32 (33.7)	58 (61.1)	35 (36.8)	28 (29.5)
Youth Aid Initiative	Ghana	NGO	53	28 (54.9)	21.18 (5.88)	N/A	8 (15.1)	40 (75.7)	5 (9.4)	0 (0.0)
UNICEF	Cambodia	iNGO	125	99 (79.2)	41.46 (11.39)	3.01 (1.45)	17 (13.6)	95 (76.0)	82 (65.6)	66 (52.8)
**Total**	**-**	**-**	**1,303**	**949 (72.9)**	**40.37 (10.47)**	**3.26 (1.90)**	**217 (16.7)**	**802 (61.6)**	**877 (67.3)**	**521 (40.0)**

^a^14 respondents did not report gender

^b^PACHAAN and Youth Aid Initiative did not report number of children; *FBO* Faith based organization, *NGO* Non-government organization, *iNGO* international non-government organization

#### Perceived impact on parenting, stress and self-efficacy

After receiving the parenting tips, 84.3% of the respondents reported that they were spending more time playing with children, 75.7% felt more capable of managing parenting stress, 79.4% reported using less physical discipline, 76.9% yelled at children less frequently, 92.2% agreed felt more confident in building positive parent–child relationships, and 85.6% had increased self-efficacy in protecting children from sexual abuse (Table 9).

**Table 9:** Perceptions of impact of COVID-19 on parenting behaviors, confidence and stress

**Agency**	**Country**	**Type**	**N**	**Play**^a^, **N (%)**	**Stress management**^b^, **N (%)**	**Physical abuse**^c^, **N (%)**	**Emotional abuse**^d^, **N (%)**	**Parenting confidence**^e^, **N (%)**	**Sexual abuse protection**^f^**,** **N (%)**
Gabriel Project Mumbai	India	NGO	166	127 (76.5)	120 (77.7)	106 (63.9)	109 (65.7)	159 (95.8)	99 (59.6)
Forgotten Voices International	Zambia	FBO	241	176 (73.0)	180 (74.7)	169 (70.1)	116 (68.9)	204 (84.6)	211 (87.6)
Forgotten Voices International	Malawi	FBO	39	38 (97.4)	36 (92.3)	33 (84.6)	33 (84.6)	39 (100.0)	38 (97.4)
Alternativa	North Macedonia	NGO	57	46 (80.7)	43 (75.4)	48 (84.2)	39 (68.4)	55 (96.5)	53 (93.0)
Karkhana	Nepal	NGO	12	7 (58.3)	8 (66.7)	9 (75.0)	6 (50.0)	10 (83.3)	8 (66.7)
Society for Promotion of Initiatives in Sustainable Development	Cameroon	NGO	129	108 (83.7)	119 (92.0)	113 (87.6)	111 (86.0)	123 (95.3)	117 (90.7)
Catholic Health Commission	Malawi	FBO	120	120 (100.0)	119 (99.2)	120 (100.0)	120 (100.0)	120 (100.0)	119 (100.0)
Alliance Development Trust	Sri Lanka	NGO	234	200 (85.5)	122 (52.1)	213 (91.0)	213 (91.0)	222 (94.9)	207 (88.5)
Protection and Help of Children Against Abuse Network (PACHAAN)	Pakistan	NGO	32	24 (75.0)	22 (68.8)	24 (75.0)	21 (65.6)	24 (78.1)	20 (62.5)
Save the Children	South Africa	NGO	95	90 (94.7)	76 (80.0)	83 (87.4)	78 (82.1)	94 (98.9)	90 (94.7)
Youth Aid Initiative	Ghana	NGO	53	50 (94.3)	43 (81.1)	42 (79.2)	39 (73.6)	48 (90.6)	42 (79.2)
UNICEF	Cambodia	iNGO	125	113 (90.4)	90 (72.0)	75 (60.0)	67 (53.6)	102 (81.6)	111 (88.8)
**Total**	**-**	**-**	**1,303**	**1099 (84.3)**	**987 (75.7)**	**1035 (79.4)**	**1002 (76.9)**	**1201 (92.2)**	**1115 (85.6)**

^a^Spending more time playing with children

^b^More able to manage stress

^c^Less physical discipline

^d^Less emotional abuse

^e^More confidence in establishing positive parent–child relationships

^f^More confidence protecting against child sexual abuse

### Differential effects by population characteristics

GLMMs were used to account for data dependency within each project while controlling for adult gender, adult age, and the number of children per caregiver. Compared to older caregivers and those with fewer children at home, younger caregivers and caregivers with more children within the household were more likely to report that they spent more time with their children (younger: OR = 0.97, 95%CI [0.95, 0.98]; more children: OR = 1.12, 95%CI [1.00, 1.24]), felt their stress management skills had improved (younger: OR = 0.97, 95%CI [0.95, 0.98]; more children: OR = 1.33, 95%CI [1.20, 1.48]), and used less physical discipline (younger: OR = 0.98, 95%CI [0.96, 0.99]; more children: OR = 1.11, 95%CI [1.01, 1.23]). Compared to older caregivers, younger caregivers reported less yelling (OR = 0.98, 95%CI [0.96, 0.99]), while older caregivers were more likely to report that the parenting tips helped them protect children from sexual abuse compared to younger caregivers (OR = 1.02, 95%CI [1.00, 1.04]). Analyses also found differences by caregiver gender, with female caregivers more likely to agree that they were using less physical discipline (OR = 0.45, 95%CI [0.33, 0.61]) and yelling less at their children (OR = 0.56, 95%CI [0.42,0.76]) than male caregivers. Parental self-efficacy in building positive parent–child relationships was similar across different demographic characteristics (Table 10).

**Table 10:** Perceptions of impact of COVID-19 by population characteristic

**Outcome**	**Caregiver gender**				**Caregiver age**				**Number of children**			
	**B**	**Sig**	**OR**	**95% CI**	**B**	**Sig**	**OR**	**95% CI**	**B**	**Sig**	**OR**	**95% CI**
**Play**	.034	.860	1.03	0.71-1.50	-0.34	< .001	0.97	0.95-0.98	0.11	.042	1.12	1.00-1.24
**Stress management**	.279	.101	1.32	0.95-1.85	-0.32	< .001	0.97	0.95-0.98	.288	< .001	1.33	1.20-1.48
**Physical discipline**	-.796	< .001	0.45	0.33-0.61	-.025	.002	0.98	0.96-0.99	.107	.031	1.11	1.01-1.23
**Emotional abuse**	-579	< .001	0.56	0.42-0.76	-.021	.007	0.98	0.96-0.99	.120	.012	1.13	1.03-1.24
**Parenting confidence**	.439	.133	1.55	0.88-2.75	-.011	.371	0.99	0.97-1.01	-0.38	.606	0.96	0.83-1.11
**Sexual abuse prevention**	-0.39	.839	0.96	0.66-1.40	0.22	.038	1.02	1.00-1.04	0.58	.317	1.06	1.10-1.23

### Maintenance

Out of the 44 implementing agencies who responded to the short surveys inquiring whether they were still using the COVID-19 parenting resources one year after initial dissemination, 14 (31.8%) shared that the resources were still on their website and 18 (40.1%) reported that they had adapted the resources for digital delivery via chatbots, apps, and social media as part of routine service delivery. Four organizations reported ongoing use of the resources in their weekly parenting sessions and visits to early childhood centers. For example, Forgotten Voices reported that they had integrated the parenting tips within programs for orphan care and faith groups in Malawi and Zambia. Likewise, the Philippine government continued to use the resources as part of their online family development services delivered as part of a conditional cash transfer program for more than four million low-income families [30]. In addition, The Human Safety Net continued to disseminate adapted versions of the COVID-19 Parenting resources as part of their yearly social communication campaigns during Parenting Month each June called ““Parenting Under Stress.” Lastly, the COVID-19 Parenting dissemination team developed new resources to support caregivers when children were returning to school and challenges around COVID-19-related bereavement – an emerging need given recent data estimating that 10.5 million children have been orphaned due to the pandemic [5].

The COVID-19 Parenting resources were also adapted for other humanitarian crises. These included adaptations for families affected by floods in Malaysia and typhoons in the Philippines in December 2021. Two years after the initial resources were developed, Parenting for Lifelong Health, one of the lead agencies in the COVID-19 Playful Parenting Emergency Response, adapted the resources for families affected by the crisis in Ukraine, which have reached an estimated 11.5 million people since 2022 [31]. The resources have also been adapted to support families affected by floods in Pakistan in 2022, recent earthquakes in Turkey/Syria and Afghanistan in 2023, and additional conflicts in Sudan and Israel/Palestine in 2023 and 2024. These adapted resources are available online available at https://www.parentingforlifelonghealth.org/crisis and via the Global Initiative to Support Parents website (https://support-parents.org/parenting-in-crisis/).

## Discussion

This study is the first of its kind to evaluate the global dissemination of a behavior change intervention aimed at supporting parents during an international crisis. It complements a parallel qualitative research study of the COVID-19 parenting resources that found high levels of perceived accessibility, cultural acceptability, and impact by implementing agencies, local communities, and parents [19]. It used the RE-AIM framework to examine their adoption, implementation, reach, effectiveness, and maintenance from a multidimensional perspective.

Findings suggest the utility of developing open-source and easily adaptable resources from evidence-based parenting programs to allow for rapid adoption, localization, and deployment. The COVID-19 Parenting response was an unprecedented collaboration with universities, NGOs, international agencies, and governments to provide immediate support to parents and caregivers during the pandemic. Their rapid adoption by 697 implementing partners was facilitated by the endorsement of UN international agencies such as the WHO, UNICEF, and UNODC, which provided both institutional legitimacy and access to a diverse network of implementers. The development of the resources by researchers from academic institutions may also have contributed to their legitimacy as parenting tips derived from “evidence-based” and “scientifically tested” programs previously evaluated in randomized controlled trials. This finding is consistent with prior research emphasizing the importance of partnerships and collaborative strategies in the successful implementation of public health interventions [32]. In particular, the adoption by 43 government agencies highlights the utility of leveraging a vast dissemination network that included individuals and organizations with pre-existing partnerships with government counterparts. The rapid adoption also underscores the importance of making evidence-based parenting intervention materials freely available and open-source through Creative Commons licensing which allowed for rapid dissemination at scale [33]. Lastly, the customization of the evidence-based parenting intervention content to the COVID-19 context may have been a critical factor of success by making sure that they were relevant to the experiences of parents across multiple settings.

In addition to the intentional development of freely available and adaptable resources, the widescale implementation of the parenting resources was also facilitated by their accessibility in local languages. The collaboration involved over 300 volunteer translators who translated the resources into 135 languages and dialects in less than a month, with expert reviewers and local implementing partners conducting quality control checks. Once provided in a local language, implementers were able to adapt the resources to fit local cultures and delivery contexts, which was essential to ensure that the resources were culturally relevant to recipients [34]. Although the most common delivery method was via social media platforms, implementation also included other innovative approaches, such as using community loudspeakers for public service announcements, caseworker toolkits, radio announcements, and faith-based guides. In addition, the availability of targeted microgrants allowed local community-based organizations to overcome implementation barriers by providing immediate funds for printing, broadcasting, or even home-based delivery. This approach aligns with literature on the adaptability and flexibility required for implementing population-level behavioral interventions, particularly in diverse cultural contexts [35].

The global reach of the COVID-19 Parenting resources represents an unprecedented level of dissemination to over 212 million individuals. This emphasizes the potential of using digital platforms to disseminate behavioral interventions at a population level [36]. It also aligns with previous research highlighting the role of social media in health communication and behavior change [37]. While the overall reach was dominated by social media, it is important to consider other platforms, such as radio, in reaching populations with limited access to the internet. Radio broadcasts have been effective in delivering health messages to remote and underserved communities, particularly in LMICs where access to digital technology is limited [38]. This combined approach, harnessing digital and traditional media along with other forms of face-to-face delivery, exemplifies the need for an integrative strategy to ensure equitable access to public health interventions [39]. Findings indicating a higher proportion of reach by geographical region in Africa and Asia may have been due to respondent biases and keyword searches primarily in English. Furthermore, although it is potential that the reach was greater in other geographical regions, it was not possible to disaggregate data from global sources that could not be attributed to a specific region or country.

Retrospective surveys across 11 countries suggested positive impacts of the COVID-19 Parenting resources. Parents reported increased parent–child play and parenting self-efficacy with decreases in parenting stress and child physical and emotional abuse. These findings are encouraging, considering global reports of increased violence against children and mental health problems during the pandemic [1, 40]. They also align with previous research on the effectiveness of in-person parenting programs in improving parent–child interactions, reducing harsh discipline, and enhancing positive parenting practices [6, 7]. Analyses exploring potential differential effects by demographic characteristics showed that younger caregivers were more likely to spend more time playing with their children and manage parenting stress effectively than older caregivers. Although this contradicts global systematic reviews indicating that parenting programs are generally as effective across parent age [7], younger caregivers may have had more access to the online parenting support due to higher levels of digital literacy. We also found gender differences, with female caregivers reporting more substantial reductions in physical and emotional abuse. This may be due to gender imbalances in caregiving responsibilities, in which female caregivers spend more time with their children and are often responsible for discipline [41]. It also corresponds to findings from a multi-country study across nine countries in which female caregivers used corporal punishment more frequently than male caregivers [42]. However, this may also be due to under-reporting by female caregivers or the fact that while male caregivers may use physical discipline less frequently, they often use more harsh forms of discipline [43]. Nonetheless, one must caution interpretation of these results due to the retrospective design (i.e., no baseline assessments), lack of a comparison group, and potential social desirability bias of self-reported responses.

While only a limited number of implementing agencies responded to follow-up surveys on the maintenance of the COVID-19 Parenting resources, results suggested their sustained delivery as they continued to be adapted and repurposed for other contexts. The adaptation of the resources to address emerging humanitarian crises is particularly encouraging considering the need for immediate parenting and child protection support during the onset of humanitarian disasters prior to the delivery of more intensive group-based programs [44]. In addition, the integration of the resources within the conditional cash transfer program for families in the Philippines suggests the importance of government adoption and institutionalization to promote positive parenting behaviors [45].

### Limitations and strengths

Several limitations are worth noting. First, reach numbers must be treated with caution since they relied on available data collected by a small, dedicated research team tracking reach across multiple platforms and self-report surveys by implementing partners. It is possible that we have overestimated reach by double-counting individuals. Even though we adopted a conservative approach that included limiting reach to one social media platform per implementing agency, individuals may have received parenting resources from multiple implementers. Likewise, estimates of absolute reach via social media and other platforms, such as radio, were operationalized based on the total number of followers or average listenership. These calculations assume passive engagement by individuals, rather than active engagement which could have been operationalized based on engagement data (i.e., likes, comments, shares). Although we considered using engagement data to calculate reach via social media, we decided against this approach since it could have potentially miss individuals who may not have actively interacted in the posts or responded to radio programs. Nonetheless, this may have overestimated the actual reach of the resources.

The use of retrospective surveys limits our ability to draw firm conclusions on the effectiveness of the resources. Data collection was limited to agencies and individuals who were willing to participate in the study, potentially introducing selection bias. Those who chose to respond may have had more positive experiences with the intervention, thus potentially overestimating its impact. Results from retrospective data may also have been imprecise due to potential recall bias and lacked a comparison group. Their generalizability is also limited since the sample only reflected a small proportion of the total reach population. In addition, retrospective surveys relied on abbreviated one-item outcome measures, which although derived from longer, psychometrically tested scales, were not validated. Lastly, low response rates to the maintenance survey limited our ability to assess the sustainability of the COVID-19 Parenting resources beyond a small sample size. To provide a more complete understanding of the intervention’s maintenance and impact over time, longer-term and more comprehensive follow-up studies are warranted [46].

Despite these limitations, this study is unique in its real-world, global scope. It provides valuable insights into the implementation of population-level behavioral interventions during the COVID-19 pandemic across several key indicators linked to implementation science. The use of multiple sources of data collection, including email and online surveys, social media metrics, and retrospective surveys with target beneficiaries, offers a rich and multifaceted understanding of real-world implementation of content derived from evidence-based parenting programs at scale. Its application of the RE-AIM framework demonstrates how implementation science methods can be used to assess the dissemination of a complex behavior change intervention during a global crisis. It underscores the pivotal role of implementation science in promoting the translation of research into practice, with potential applications in various domains, including public health, global health, community-based interventions, child development, and climate response.

### Implications for future research and practice

The findings of this study have several implications for future research and practice. First, it underscores the need for continued research to build a stronger evidence base for the effectiveness and maintenance of population-level parenting support interventions during public health emergencies. It provides a blueprint on how to adapt evidence-based interventions into applied resources and underscores the vital importance of allocating sufficient resources to conduct evaluations during dissemination. Although challenging to conduct in times of acute crisis, more robust research designs, including randomized trials, are essential for establishing causal relationships between the use of resources and the observed outcomes. The trial of *Parent Positive*in the United Kingdom is an innovative example in which researchers were able to rapidly develop a parenting app and test it using a randomized design during the pandemic by leveraging an existing cohort study [47]. Second, the study highlights the importance of inter-agency collaboration at both national and global levels to effectively address public health crises. The engagement of a diverse range of implementing agencies, including governments, NGOs, faith-based organizations, and academia, demonstrates the ongoing need for collective efforts to reach and support families during crises. Third, the study suggests the potential of diverse platforms for dissemination, such as radio and community broadcasts, particularly in regions with limited internet access [13]. Finally, this study highlights the benefits of making evidence-based interventions freely available so that they can be widely adopted and customized for local populations [33].

## Conclusion

The COVID-19 pandemic posed unprecedented challenges for families worldwide, particularly affecting those in low- and middle-income countries and marginalized communities. The COVID-19 Playful Parenting Emergency Response was part of a much larger global effort to support parents and caregivers who were on the frontlines of the pandemic. This study demonstrates the feasibility and success of implementing population-level behavioral interventions during global crises. Guided by the RE-AIM framework, results provide valuable insights into the adoption, implementation, reach, effectiveness, and maintenance of the COVID-19 parenting resources. Findings offer important lessons for future research and practice in the fields of implementation science and global emergency responses, helping us understand how behavioral interventions can be integrated into real-world practice and policy, particularly during exceptional circumstances like a global pandemic.

## Data Availability

The datasets used and/or analyzed during the current study are available from the corresponding author upon reasonable request.

## Abbreviations

COVID-19: SARS-CoVCoV-2
FBO: Faith-based organization
GLMMs: Generalized linear mixed models
LMICs: Low-and middle-income countries
NGO: Non-governmental organization
OR: Odds ratio
PLH: Parenting for Lifelong Health
RE-AIM: Reach, Effectiveness, Adoption, Implementation, and Maintenance
UNICEF: United Nations Children’s Fund
UNODC: United Nations Office on Drugs and Crime
WHO: World Health Organization
